# Insights into dynamin-associated disorders through analysis of equivalent mutations in the yeast dynamin Vps1

**DOI:** 10.15698/mic2016.04.490

**Published:** 2016-03-22

**Authors:** Laila Moustaq, Iwona I. Smaczynska-de Rooij, Sarah E. Palmer, Christopher J. Marklew, Kathryn R. Ayscough

**Affiliations:** 1 Department of Biomedical Science, University of Sheffield, Sheffield, S10 2TN, UK.

**Keywords:** Dynamin, Charcot-Marie-Tooth, Epilepsy, Disease mutation, Saccharomyces cerevisiae

## Abstract

The dynamins represent a superfamily of proteins that have been shown to function in a wide range of membrane fusion and fission events. An increasing number of mutations in the human classical dynamins, Dyn-1 and Dyn-2 has been reported, with diseases caused by these changes ranging from Charcot-Marie-Tooth disorder to epileptic encephalopathies. The budding yeast, *Saccharomyces cerevisiae* expresses a single dynamin-related protein that functions in membrane trafficking, and is considered to play a similar role to Dyn-1 and Dyn-2 during scission of endocytic vesicles at the plasma membrane. Large parts of the dynamin protein are highly conserved across species and this has enabled us in this study to select a number of disease causing mutations and to generate equivalent mutations in Vps1. We have then studied these mutants using both cellular and biochemical assays to ascertain functions of the protein that have been affected by the changes. Specifically, we demonstrate that the Vps1-G397R mutation (Dyn-2 G358R) disrupts protein oligomerization, Vps1-A447T (Dyn-1 A408T) affects the scission stage of endocytosis, while Vps1-R298L (Dyn-1 R256L) affects lipid binding specificity and possibly an early stage in endocytosis. Overall, we consider that the yeast model will potentially provide an avenue for rapid analysis of new dynamin mutations in order to understand the underlying mechanisms that they disrupt

## INTRODUCTION

Dynamin belongs to a conserved family of large GTPases involved in a wide range of cellular processes including vesicle trafficking, vesicle scission, synaptic vesicle recycling, organelle division, viral resistance and cytokinesis 1,2,3. The importance of dynamin was first discovered in *Drosophila melanogaster*, where mutations in the *shibire* gene caused paralysis at high temperature by reversibly blocking endocytosis at nerve terminals 4,5. Classical dynamin proteins are characterized as having five domains: an N-terminal GTPase domain involved in nucleotide hydrolysis, a middle domain and a GTPase effector domain (GED) both important in oligomerization, a pleckstrin homology (PH) domain involved in binding membrane lipids 6,7, and a C-terminal proline rich domain (PRD) which contains sites for binding the Src-homology 3 (SH3) domain of accessory proteins 3,8. In mammals, 3 classical dynamin genes are present, coding for Dyn1, Dyn2 and Dyn3. Dyn1 is expressed highly in neurons, Dyn2 is expressed ubiquitously and Dyn3 is expressed in the brain but at much lower levels than Dyn1, as well as in the testis and the lungs 9. Dyn1 has been most extensively studied, and is generally considered to function as a mechanochemical enzyme to bring about constriction and fission of vesicles during endocytosis 3,10. However, dynamin proteins have also been implicated in other processes, including cytokinesis, export from the Golgi, caveolae dependent endocytosis, macropinocytosis and autophagy 1,11,12,13,14,15.

The emerging realisation that dynamins can function at multiple membranes rather than just at the plasma membrane in endocytosis raises questions as to how specificity for its fission functions can be achieved and which proteins are able to regulate their function at distinct compartments.

A number of missense mutations and short deletions in the middle or the PH domain in Dyn2 have been associated with two autosomal dominant genetic disorders, Charcot-Marie-Tooth disorder (CMT) and Centronuclear myopathy (CNM) 11,16,17,18. Interestingly, the diseases caused by the mutations are exclusive indicating that while mutations may lie in the same domain, they can impact differently on protein function. An additional Dyn2 mutation G358R, that gives rise to CMT disorder, lies outside the PH domain within the highly conserved stalk region of the dynamin protein.

Until very recently, no human disease mutations have been reported in Dyn1 or Dyn3, however, a missense mutation (A408T) in Dyn1, has been shown to give rise to the fitful mouse. This mutation results in seizures and hearing impairment and was suggested as a candidate for epilepsy in humans 19. Exercise Induced Collapse (EIC) condition in Labrador Retrievers is another condition mapped to Dyn1, in which an R256L missense mutation in the GTPase domain of Dyn1 resulted in acute and severe muscle weakness leading to life threatening collapse of the animal as a result of intense exercise or excitement 20. Recently three mutations have been reported in human Dyn1 that give rise to epileptic encephalopathy, these are A177P, K206N and G359A 21. It is notable that the last mutation lies adjacent to the CMT mutation mentioned above, G358R.

There are no classical dynamin proteins present in yeast. However, there are three dynamin-like proteins, Dnm1, Mgm1 and Vps1, all of which contain the N-terminal GTPase domain, the middle domain and the GED domain. While Vps1 is involved in membrane trafficking, Dnm1 is involved in mitochondrial fusion and fission and Mgm1 plays a role in maintaining the mitochondrial genome and inheritance 22,23. Vps1, like Dyn-2, has been shown to be involved in a number of membrane fusion/fission processes, including trafficking from the Golgi, endosomal trafficking and endocytosis 24,25,26,27,28. Taken together, these data suggest that Vps1 is functioning in a similar way to classical dynamins in mammalian cells.

This study aimed to use Vps1 as a model to gain insights into the mechanistic defects caused by specific dynamin mutations considered to underlie a number of diseases.

## RESULTS

### Generation of orthologous disease mutations in Vps1.

**Figure 1:**
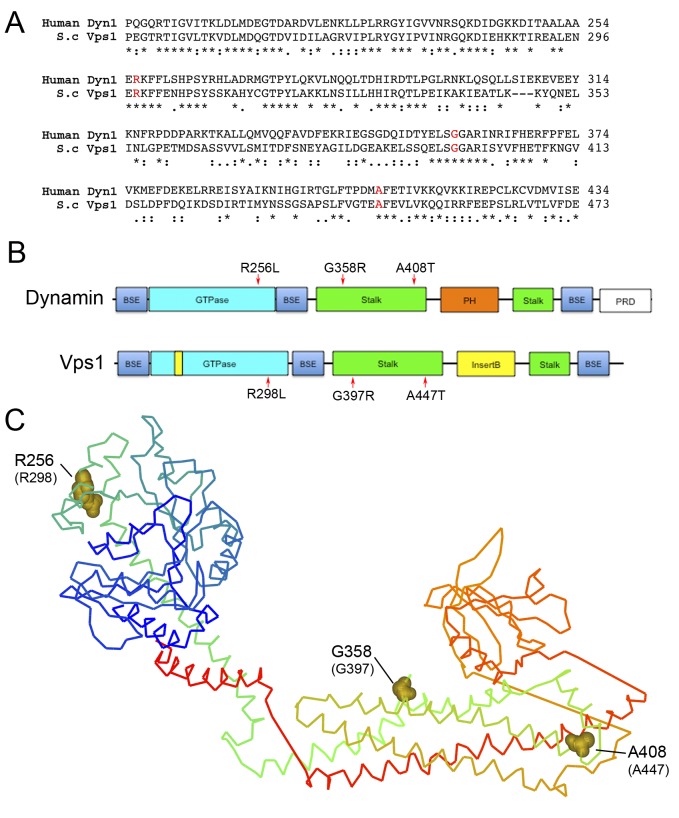
FIGURE 1: Location of Dynamin Mutations. **(A)** Sequence alignment of relevant region of Dyn-1 and Vps1 showing the position of amino acids selected for mutation. Accession numbers Human Dynamin-1 AAH50279; Vps1 CAA82071. **(B)** Schematic showing domain structure of mammalian dynamin and yeast Vps1 and corresponding positions of mutations. BSE - bundle signaling element, PH - pleckstrin homology, PRD - proline rich domain. **(C)** The crystal structure of Dynamin-1 (PDB number 3SNH) with dynamin-1 positions denoted with the equivalent mutagenized residues in Vps1 in parentheses.

Three equivalent mutations known to cause different diseases in mammals were introduced into the *VPS1* gene. These were the Dyn1 mutations R256L (Vps1-R298L) causing exercise-induced collapse in Labradors, A408T (Vps1-A447T) that causes epilepsy in the fitful mouse model, and the Dyn2 mutation G358R (Vps1-G397R) which causes CMT disorder 19,20,29. The sequence alignment of dynamin and Vps1, and the positions of the mutations are shown in Figure 1A. The mutation positions are also shown in a schematic diagram, indicating Vps1-Dynamin domain structure, and in the equivalent places on the dynamin crystal structure (Figure 1B, C) 30,31. The R256L mutation lies in a highly conserved region of the N-terminal GTPase domain. In the crystal structure this residue is exposed and does not form part of the GTP binding pocket required for GTPase activity. The A408T mutation lies within the stalk region at one end of a helix that has been shown in both Dyn1 and Vps1 to bind to actin 32,33. The position of the G397 mutation also maps to a highly conserved stretch of residues on the stalk domain. This part of the protein is considered to be important for oligomerization 30,31,34.

### Expression of Disease mutations in Yeast cells.

**Figure 2:**
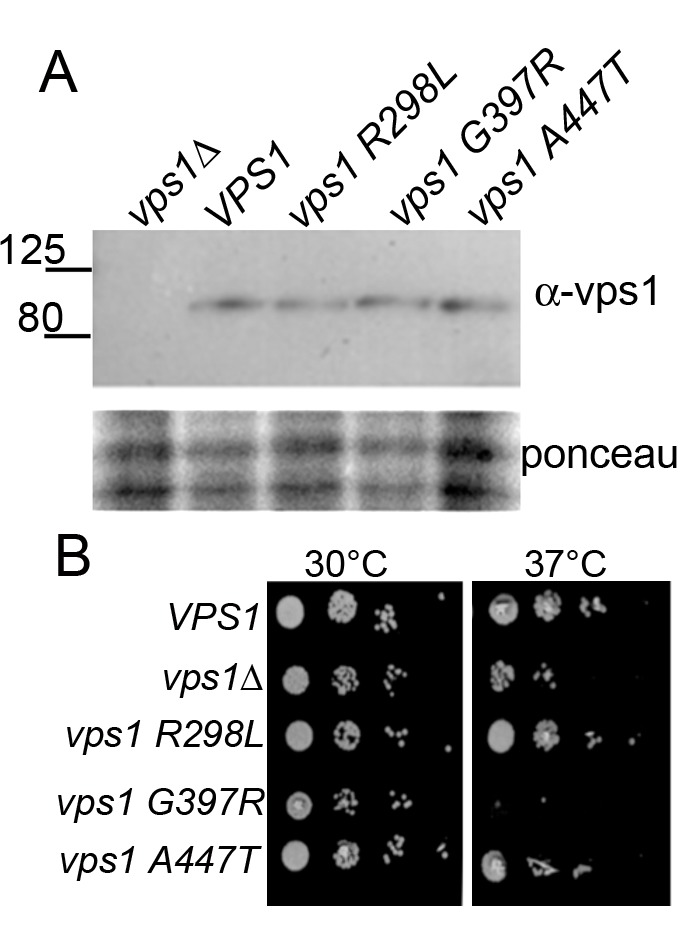
FIGURE 2: Expression of mutant Vps1 in yeast. **(A)** Whole cell extracts were made from *vps1* null cells expressing an empty plasmid, wild type or mutant *vps1*. Following separation by SDS-PAGE, proteins were transferred to PVDF, then probed with anti-Vps1 antibodies. Size markers in kDa are denoted. Lower panel shows ponceau staining of the blot to indicated loading levels in lanes. **(B)** The effect of *vps1* mutants on cell growth was assessed by growth on solid media. Ten-fold serial dilutions of log phase cultures were spotted onto plates, and cells were allowed to grow at the permissive (30°C) or restrictive temperature 37°C) for 48 hours.

The *vps1* mutations were generated in a yeast expression plasmid in which *VPS1* is expressed under the control of its own promoter. The plasmids were transformed into yeast carrying a deletion of the *VPS1* gene, ensuring no endogenous protein was present. Expression levels of Vps1 were determined by western blotting using antibodies against Vps1. As shown (Figure 2A) the wild type protein and its mutant variants are expressed at similar levels in cells.

We then sought to determine whether the mutant forms of *vps1* affect cell growth. *vps1* null cells carrying an empty plasmid, wild type *VPS1*, or one of the mutants were spotted onto plates to assess growth. Deletion of *vps1* caused a temperature sensitive phenotype at 37°C. As shown in Figure 2B, two mutants, *vps1-R298L* and *vps1-A447T,* were able to rescue the temperature sensitivity associated with *vps1* deletion. However, the *vps1-G397R* mutant did not rescue the temperature sensitive phenotype of *vps1* deletion, indicating that this mutation might cause a loss of function despite being expressed at wild-type levels.

### Analysis of membrane functions in the Vps1 mutant strains.

**Figure 3:**
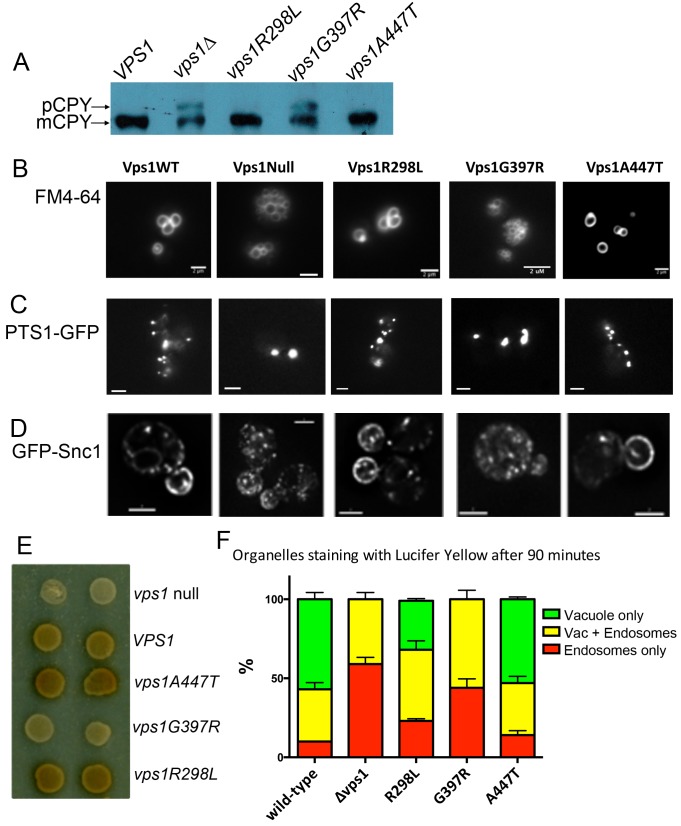
FIGURE 3: The effect of Vps1 mutations on functions requiring Vps1. **(A)** Whole cell extracts were made from yeast expressing mutated *vps1* as the only form of Vps1. These were separated by SDS-PAGE, transferred to PVDF membrane and probed with anti-CPY antibodies. Two bands are observed, the mature protein (mCPY) and the immature, precursor form (pCPY). **(B)** The lipophylic dye FM4-64 was used to determine whether the mutations affect vacuolar morphology. **(C)** Cells were transformed with a peroxisomal targeting sequence fused to GFP. This allowed the morphology of peroxisomes to be visualized in strains lacking *vps1* and *dnm1* but re-transformed with *VPS1* wild type or mutants. **(D)** A strain was generated expressing GFP-Snc1-*SUC2* that allows endosomal cycling to be investigated. GFP indicates localization of this reporter. **(E)** The same GFP-Snc1-*SUC2* reporter also allows invertase activity present at the cell surface to be detected in a colorimetric assay on plates as described in text. **(F)** Endocytic uptake into cells was monitored using Lucifer yellow which was incubated for 90 min with all strains. The predominant localization in 100 cells of each strain, in 3 independent repeats was counted. Error bars are standard error of the mean.

As mentioned above, Vps1 function is required in a number of membrane remodeling events in cells, including trafficking of the vacuolar protein carboxypeptidase Y (CPY) from the Golgi to vacuoles 35,36. Deletion of *vps1* also causes a class F vacuolar phenotype with multiple, small or heterogeneous vacuolar structures 35. To determine whether the mutations impacted on the functions of Vps1 required for these roles, cells expressing wild type *VPS1*, *vps1* mutants or a *vps1* deletion were analysed for CPY trafficking. If CPY is able to reach the vacuole, the CPY enzyme is processed by cleavage of precursor forms and a mature form is generated. If, however, CPY is not effectively trafficked to the vacuole, as in the *vps1* deletion strain a precursor form can be visualized on a blot (Figure 3A). Two of the mutants *vps1-R298L* and *vps1-A447T* were able to rescue this trafficking defect and only mature CPY is seen on the blots. The G397R mutant, however, was not able to rescue the trafficking defect and the precursor CPY form was still clearly visible (upper arrow).

The lipophylic dye FM4-64 was then used to analyze vacuolar morphology in the mutants. As shown in Figure 3B, in wild type cells there are a small number of vacuoles, usually 3-5, of a uniform size. In contrast, the *vps1* null strain has many small vacuoles. Again the mutants have distinct phenotypes with the *vps1-R298L* and *vps1-A447T* mutants having vacuolar morphologies similar to the wild type strain and the *vps1-G397R* mutant similar to the deletion.

Vps1 has also been demonstrated to function in fission of peroxisomes 37. These organelles grow and divide by a fission mechanism to ensure appropriate inheritance. In wild type cells there are usually multiple small peroxisomes distributed throughout the cytoplasm. In the absence of both dynamins *vps1* and *dnm1* there is a phenotype in which the number of peroxisomes is greatly reduced (Figure 3C) 37. This phenotype can be completely rescued by re-expression of Vps1. As shown the mutants *vps1-R298L* and *vps1-A447T* rescued the fission defect of peroxisomes but the *vps1-G397R* mutant did not.

The effect of the *vps1* mutants on endosomal trafficking was investigated. Previously, we have shown that deletion of *vps1* disrupts normal trafficking of a reporter construct, containing the SNARE protein Snc1 fused to both GFP at its N-terminus and *SUC2* conferring invertase activity at its C-terminus 38. This colorimetric assay allows differentiation between endosomal trafficking and endocytic internalisation defects 38,39. If Vps1 function is predominantly intracellular, for example in endosomal fusion/fission events, then its loss will result in the Snc1-invertase fusion accumulating inside cells, so when cells are incubated with appropriate substrate colonies will be white. If the primary function of the protein is in endocytic internalization, then uptake of the SNARE will be delayed, more invertase will be exposed at the surface and in the assay colonies will appear darker brown. In wild-type cells there is continuous cycling of Snc1, and the cells when assayed appear light brown. Cells in which *vps1* was deleted were transformed with an empty plasmid or with plasmids carrying wild-type or mutant *vps1*. Both localization of the GFP and the production of invertase were assayed.

As previously shown, in *vps1*∆ cells the GFP reporter is predominantly in internal vesicles while in wild type cells the reporter localizes mostly to the plasma membrane and to brightly staining internal organelles 25. The G397R mutation causes a localization phenotype similar to the null, while the R298L and A447T mutations have high levels of plasma membrane Snc1 localization, similar to wild type Vps1. Invertase at the surface was also assessed and, as shown in Figure 3E, the null strain is white and the cells expressing wild-type *VPS1* develop a light brown color. As found in the assays described above, the G397R phenocopies the null, revealing a defect in endosomal recycling in cells expressing this mutation. In contrast, cells carrying the R298L mutation or the A447T mutation appear slightly darker brown than cells with wild-type *VPS1*, indicating that while other functions appear normal, there may be a defect in endocytic uptake.

To further investigate this phenotype, the effect of the mutations on endocytic uptake and trafficking to the vacuole was followed using the dye Lucifer yellow. After 90 minutes of incubation with Lucifer yellow the majority of cells with wild type *VPS1* have a labeled vacuole while the predominant phenotype in the *vps1* deletion is endosomal staining (Figure 3F). As expected from the other assays the G397R mutation caused a similar extent of defect compared to the deletion strain. However, in this assay the R298L mutant, and to a lesser extent the A447T mutant, caused effects on trafficking of Lucifer yellow dye to the vacuole.

Taken together these assays indicate that the G397R mutation has phenotypes resembling the null strain. Thus, while the *vps1-G397R* protein is expressed, we would suggest that it is largely non-functional. Intriguingly, the other two mutants, R298L and A447T, appeared to function at a level similar to the wild-type protein for the majority of Vps1 roles. Only in assays measuring endocytic activity were deficits were observed.

### Effect of *vps1* mutations on individual endocytic events.

**Figure 4:**
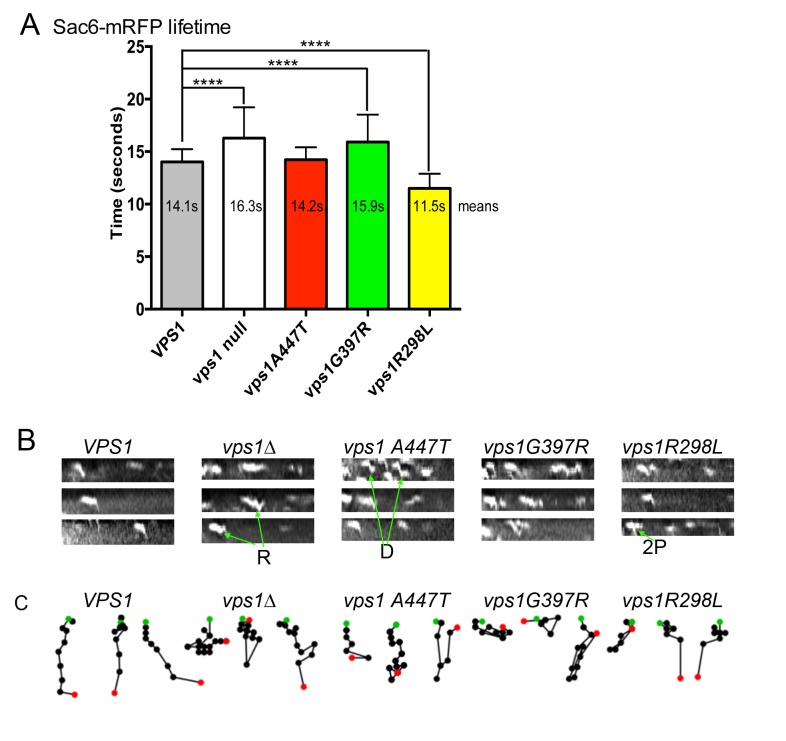
FIGURE 4: The effect of Vps1 mutations on individual endocytic events. Cells expressing Sac6-mRFP and deficient for *vps1* were transformed with an empty plasmid and wild type or mutant *vps1*. **(A)** Time-lapse movies were recorded and used to measure the lifetime of the reporter at endocytic sites. Means are reported, error bars are standard deviation. A 1-way-Anova was used with Dunnett’s multiple comparison test to assess significance of differences from the wild type. Those marked with asterisks are considered significant p≤0.0001. **(B)** Movies were also used to generate kymographs for each of the strains to analyze the behavior of patches during invagination. Indicated with arrows: R - retraction event, D- delayed scission/disassembly event, 2P - second patch event. **(C)** Patch tracks were generated to follow the movement of patches. Three are shown for each strain with the center of each spot marking the recorded position at each time point. Green spot indicates the start point and red the last point before disassembly.

In other studies we have observed abnormal endocytic phenotypes caused by *vps1* alleles in cells that appear to carry out other Vps1 functions appropriately 25,33,40. We therefore aimed to determine whether any of the disease mutations were able to affect the progression of individual endocytic events. Wild type and mutant *vps1* alleles were expressed in cells carrying the mRFP tagged endocytic reporter Sac6. Sac6 is an actin bundling protein required for normal invagination of the membrane at the endocytic site. The lifetime of Sac6-mRFP at the endocytic sites was measured in cells expressing wild type or mutant *vps1*. In addition, kymographs were generated and patch tracking was performed using time-lapse movies of the Sac6-mRFP reporter to investigate possible changes in invagination behavior. As noted before, and shown in Figure 4A, in the absence of *vps1* there is an increase in lifetime of the Sac6 reporter 40. As expected, the G397R mutation, that phenocopied the null strain in other assays (Figure 3), also showed a similar significant increase in Sac6-mRFP lifetime. In contrast the A447T mutation did not significantly affect lifetime of Sac6 at the site, and the lifetime of Sac6-mRFP in cells expressing *vps1-R298L* was in fact reduced compared to cells with wild-type *VPS1*. The patch tracking and kymograph data (Figure 4B, C) provided further insights into the endocytic defect of cells expressing these mutations. As shown before, the majority of patches in wild type cells show clear invaginations while there are frequent retraction events in cells lacking *vps1*. In the case of *vps1-A447T* the majority of patches showed invagination but there were often delays in scission as indicated by the Z-shaped kymographs indicative of inward movement followed by a pause before disassembly. The R298L behaviour was distinct from those in cells expressing either wild type *VPS1* or *vps1-A447T*. The majority of events were short-lived, but invagination was difficult to discern as sometimes movement was not perpendicular and often a second patch appeared to underlie the first (denoted 2P) in figure. Similar to cells with wild type *VPS1* retraction events in cells with R298L were much less frequent (<30% of events) than in the cases of the other mutants.

One possible reason for lack of function in endocytosis is that the mutant proteins simply don’t localize to the appropriate site. However, our antibodies have not proved effective for immunofluorescence microscopy. In addition we and others have noted that GFP tagging of Vps1 renders the protein non-functional 33,41. However, when such endocytic defective *vps1* mutants have been tagged, co-localization with endocytic markers has been demonstrated 33,40,42. Furthermore, the fact that both the A447T and R298L proteins generate distinct phenotypes from one another and from the null strain lead us to consider that the ability to be recruited to the endocytic site is very likely to be maintained.

### Effect of mutations on biochemical properties of Vps1.

**Figure 5:**
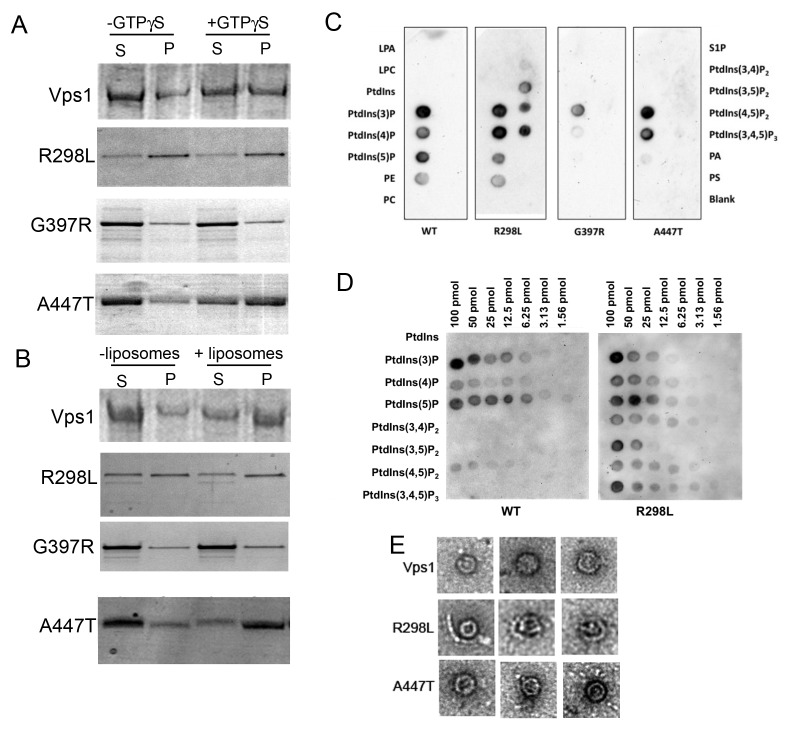
FIGURE 5: Biochemical analysis of Vps1 mutant proteins. His-tagged versions of the wild type and mutant Vps1 proteins were purified as described in the materials and methods. **(A)** Protein was incubated in the presence and absence of GTPγS which can lock the protein in the GTP bound form and stabilize the oligomeric form. Following centrifugation supernatant (S) and pellet (P) fractions were run on a gel and stained with Coomassie blue stain. **(B)** Protein was incubated in the presence and absence of liposomes. Samples were centrifuged to determine ability to interact with lipids and pellet with the liposomes. Following centrifugation supernatant (S) and pellet (P) fractions were run on a gel. Proteins were stained with Coomassie. **(C)** Binding to specific lipids was investigated using a PIP strip membrane approach. Proteins were incubated as described and binding assessed by western blotting. **(D)** A PIP array was used to further address the wider binding specificity of the R298L mutant compared to wild type Vps1. **(E)** Purified proteins were also analyzed following negative staining using electron microscopy. Representative oligomeric rings of Vps1 are shown.

To further improve our understanding of the molecular functions affected by the mutations, biochemical assays were performed. Following purification, the proteins were assayed for their ability to oligomerize and thereby pellet in the presence of GTPγS. As shown previously and in figure 5A, addition of GTPγS allows stabilization of oligomers and there is a concomitant shift of wild type protein into the pellet fraction (P) 25. The R298L mutation appears to induce either aggregation, or more stable oligomer formation irrespective of GTPγS addition and the purified Vps1-R298L protein pellets in both cases. The G397R mutation appears to reduce oligomer assembly and there is no shift into the pellet fraction when GTPγS is included. A447T mutation does not affect the ability of Vps1 to oligomerize and a shift to a more stable oligomeric form can be induced by addition of GTPγS.

Vps1 has previously been shown to interact with lipids and to pellet with liposomes. Thus a liposome-binding assay was used to determine whether the mutations compromised this function. Incubation with liposomes was followed by centrifugation to determine binding (Figure 5B). Wild type Vps1 is observed to shift into the pellet fraction in the presence of liposomes indicating lipid binding. As before, the majority of *vps1-R298L* pellets regardless of the presence of liposomes. The G397R mutant does not appear to move into the pellet fraction, suggesting the lipid binding is compromised in this mutant. A447T mutants showed a shift into the pellet fraction in the presence of liposomes, indicating that lipid binding is maintained. Because of problems with protein pelleting in the absence of GTPγS or liposomes, especially in the case of the R298L mutant, another approach was used to investigate lipid binding without the need for centrifugation. This approach investigated protein binding to lipids spotted on membranes. These PIP binding assays were carried out as described in materials and methods. As shown, the wild type Vps1 is able to interact with lipids in this format, with greatest binding detected with phosphatidyl inositol monophosphates. The A447T mutant gave a broadly similar pattern of binding to the wild type protein. A reduction in PI5P binding was observed but the physiological consequence of this is not clear as PI5P is considered to be a relatively rare PI monophosphate lipid found in the nucleus 43. In contrast, the G397R mutation showed greatly reduced binding to these PIP strips, supporting the centrifugation data showing reduced pelleting with liposomes (Figure 5C). Intriguingly, it was notable that the R298L mutant seemed to alter lipid binding specificity such that a broader range of bis-phosphatidylinositol lipids could now be bound in addition to the mono-phosphates. This broader spectrum of lipid binding was borne out by a more quantitative PIP array assay in which a dilution series of each phosphatidyl inositol phosphate was spotted onto the membrane (Figure 5D). 

To address possible differences in oligomerization capability electron microscopy of protein samples was performed. We recently demonstrated that wild type Vps1 protein is able to form ring structures 33. Proteins were purified and applied to grids as described. Ring structures were observed for wild-type protein and the R298L and A447T mutants but could not be observed for *vps1-G397* (Figure 5E). The average size of the single rings formed was 31.6±2.9 nm for wild-type Vps1; 26.3±2.9 nm for A447T and 29.0±3.3 nm for R298L mutant proteins. The wild type and *vps1-A447T* rings were significantly different in size assessed using a non-parametric t-test (p=0.0025). Neither the A447T nor the R298L mutant was observed to form double ring structures that have previously been observed for wild type protein 33.

## DISCUSSION

In this study three Vps1 mutations were generated to mimic the equivalent dynamin mutations that cause neurological diseases in mammals. The effect of each mutation was analyzed in several *in vivo* assays. In addition, recombinant mutant proteins were expressed and purified, and the effect of the mutations on protein behavior was studied biochemically. Importantly all three mutants could be expressed in yeast cells at levels similar to that in the wild-type, indicating that none of the mutations caused a major disruption of protein folding and stability.

The various assays undertaken indicate that different functions are affected in each of the mutants. The mutation that caused the greatest effect on protein function was the G397R mutation. This is equivalent to G358R in human dyn-2 that has been associated with CMT disorder. This mutation introduces a large, basic residue in place of a small residue that often confers flexibility to regions of proteins. Both the charge and the reduced flexibility might have a marked impact on function. The region which carries this mutation is highly conserved from yeast to mammalian Dyn-1 with 100% identity in the motif (352 ELSGGARI 362; Figure 1). The motif lies along the stalk region known to be important for oligomerization of dynamins 30,31. It was therefore not completely surprising that this mutation had a negative effect on oligomerization *in vitro* (Figure 5). Interestingly, the mutation also caused a dramatic reduction in lipid binding. It has been reported that oligomeric association of dynamin monomers is important for lipid binding by the dynamin PH domain 44. The result observed for Vps1 G397R mutation in this study would suggest that oligomerization is also important for Vps1 lipid binding, thus leading to the notion that the primary defect in the G397R (G358R) mutant is likely animpaired oligomerization. One intriguing conclusion is that a mutation in the same GG motif can cause epilepsy in Dynamin-1, and CMT disorder in Dynamin-2 . Given that the mutation essentially appears to completely inhibit function of the protein through disrupting oligomerization, the diseases presumably reflect the underlying function of the different dynamin proteins within certain tissues. 

The A447T mutation had a more subtle effect on the overall function of Vps1 than the G397R mutation. Although neither the wild-type nor the mutant residues have a charge, threonine is a slightly larger residue with a hydroxyl group that could participate in hydrogen bond interactions, which may disturb the protein conformation. While this mutation also lies in the stalk region, unlike the G397R mutation, it did not affect the oligomerization of the protein neither did it affect the lipid binding. In fact, the majority of *in vivo* assays performed revealed a phenotype similar to the wild-type, except for endocytosis where the mutation lead to reduced trafficking of Lucifer yellow and of the GFP-Snc1-Invertase construct from the plasma membrane. Analysis of the behavior of the endocytic reporter Sac6-mRFP indicated a striking phenotype with most patches showing invagination but ensuing defects in reporter disassembly from the site presumably due to a problem in the final scission stage. 

The A408T (fitful) mutation is known to disrupt transferrin uptake in mammalian cells 19; our unpublished data and from their cross-linking studies it was reported that the protein was less able to form dimers and tetramers. This latter result would appear to conflict with the data for Vps1-A447T with the mutant protein showing normal levels of oligomerization. In fact, in our electron microscopy experiments we observed a shift towards the oligomerized ring state, such that after purification, ring structures were easily visualized and more prevalent in electron microscopy analysis for *vps1-A447T* than with Vps1 from wild-type cells. Because the mutation lies at one end of the actin binding helix 32,33 we also investigated whether the mutation affected binding to filamentous actin. However, no significant difference was noted (data not shown). The reason for the discrepancy over the effect of the mutation on oligomerization state could be determined through further investigation of the A408T protein. In the reporter study only the monomer, dimer and tetramer state were investigated after cross linking; it remains a possibility that if a more stable, higher order structure had been generated, it would probably not enter the gel and therefore, would not be as readily analyzed. Use of electron microscopy would also clarify whether oligomeric rings or spirals of A408T Dyn1 can still be generated.

The R298L mutation led to a large positive hydrophilic residue being substituted for an uncharged hydrophobic residue. This removal of charge might have been expected to have adverse effects on protein-protein interactions. Although the R298L mutant protein was successfully purified, the protein appeared to have a greater tendency to form aggregates, which made interpretation of some *in vitro* work problematic. As a result, it was difficult to conclude whether this mutation affects the oligomerization of the protein. The electron microscopy analysis indicated that the protein could still form rings, though the rings seemed more variable in size and shape than observed with an equivalent concentration of the Vps1 wild type or *vps1-A447T* incubated under the same conditions. Intriguingly, the PIP strip analysis revealed significant changes in the lipid binding specificity of R298L. Since this mutation lies in the GTPase domain, it was unexpected for an effect on lipid binding to occur, as this function has been suggested to lie within the region in the protein equivalently placed to the PH domain (InsertB). In the cellular assays, the majority of functions were judged to be performed similar to wild-type with the exception of endocytosis. In this case the defect appeared to be due to early disassembly of the reporter protein, suggesting that the endocytic site was somehow not established appropriately for effective invagination. The defect however appears to be at an earlier stage and quite distinct to that caused by the A447T mutation, as in the case of R298L, retractions and delayed scission was not observed.

This suggests that the change of the lipid binding affinity might be more important for the function of the protein during endocytic scission while other functions are less affected by this broadening of binding specificity. One of the recently identified human Dyn-1 mutations (A177P) also lies in the N-terminal GTPase domain. As with the N-terminal Vps1 mutation, this mutation does not affect oligomerization but does affect uptake of transferrin. It will be of interest to determine whether lipid-binding properties of this human mutation are also affected.

Overall we have shown that the mutation at equivalent sites of Vps1 to Dyn-1 and Dyn-2 mutations can be used to model fundamental aspects of dynamin function. Identification of novel human mutations such as A177P and K206N also opens the door to further use *S. cerevisiae* as a model system to increase our understanding of the stage of function that these mutations affect endocytosis or other dynamin functions in cells. Given the ability of the *vps1-A447T* and R298L proteins to selectively affect endocytosis but not other Vps1 functions raises interesting questions as to how certain dynamin functions can be selectively inhibited and may help to shed light on these factors as new roles for dynamins in mammalian cells emerge.

## MATERIALS AND METHODS

### Materials

Unless stated otherwise, chemicals were obtained from Sigma-Aldrich (St Louis, Missouri). Media was from Melford Laboratories, Ipswich, Suffolk, UK (yeast extract, peptone, agar) or Sigma (minimal synthetic medium and amino acids). FM4-64 was from Invitrogen; Lucifer Yellow from Fluka.

### Yeast strains and cell growth

**Table 1:** Yeast strains used in this study.

**Strain number**	**Genotype**	**Origin**
KAY447	*MATα his3∆1, leu2∆0, lys2∆, ura3∆0*	KA lab
KAY1095	*MATα his3∆1, leu2∆0, lys2∆, ura3∆0 ∆vps1::KanMx*	KA lab
KAY1096	*MATα his3∆1, leu2∆0, lys2∆, ura3∆0 ∆dnm1::KanMx ∆vps1::HIS5*	E.Hettema (Univ of Sheffield)
KAY1368	*MATα Sac6-RFP::KanMX ∆vps1::Leu*	KA lab
KAY1462	MAT*α his3∆1, leu2∆0, lys2∆, ura3∆0 ∆vps1::KanMx*, *GFP-Snc1-SUC2* *URA3*	KA lab

Yeast strains used in this study are listed in Table 1. Cells were grown with rotary shaking at 30°C in liquid YPD medium (1 % yeast extract, 2 % Bacto-peptone, 2 % glucose supplemented with 40 µg/ml adenine) or in synthetic medium (0.67 % yeast nitrogen base, 2 % glucose) with supplements. Transformations were performed using lithium acetate as described 45. Point mutations in *VPS1* gene were generated using site directed mutagenesis (QuikChange Lightning kit, Agilent) with plasmids pKA677, and pKA850 as the templates. The constructs were then verified by sequencing. Plasmids are listed in Table 2. Vps1 protein was detected on western blots of whole cell extracts using anti-Vps1 antibody (1:2000 dilution). Carboxypeptidase Y processing was analysed from cell extracts as described 40. Pre-cleaned CPY antibodies (Chemicon International) were used at 1:100 dilution.

**Table 2:** Plasmids used in this study.

**Plasmid number**	**Description**	**Origin/Reference**
pKA544	URA, CEN with PGKterm	KA lab
pKA677	pKA544+ *VPS1* (inc 320bp 5’)	25
pKA798	pKA544+ *vps1* R298L	This study
pKA797	pKA544+ *vps1* G397R	This study
pKA796	pKA544 + vps1 A447T	This study
pKA850	His tagged VPS1 for recombinant protein expression	40
pKA819	pKA850 + *vps1* R298L	This study
pKA820	pKA850 + *vps1* G397R	This study
pKA821	pKA850 + *vps1* A447T	This study
pKA910	GFP-SKL (peroxisome marker) *URA*	Hettema lab

### Cell Biology

Epifluorescence microscopy was performed using Olympus IX-81 microscope with DeltaVision RT Restoration Microscopy with 100x 1.40 NA oil objective and Photometrics Coolsnap HQ camera. Imaging and image capture was performed using SoftWoRxTM (Applied Precision Instruments, Seattle). Experiments were carried out at 21°C. For uptake of FM4-64, 0.25 μl of 16 mM FM4-64 was added to 500 μl culture for 90 minutes. Following washing, Z-stack images were collected with step sizes of 0.2 µm.

Lucifer Yellow uptake: Cells were incubated with Lucifer yellow (Fluka: 13 mg/ml final concentration) for up to 90 mins. Cells were washed in buffer (50 mM succinate, 20 mM NaN_3_ pH 5) before imaging. For peroxisomal fission: cells were transformed with GFP-PTS1 46. For live-cell imaging, cells were visualized in synthetic medium. Time-lapse live cell imaging of Sac6-mRFP was performed with 1 sec time-lapse and 0.5 sec exposure.

### Biochemical Approaches

Wild type *VPS1* and *vps1* mutants were expressed in *E. coli* (C43) (Lucigen OverexpressTM C43(DE3) SOLOs) as His tag fusions and were purified as previously described 25. Self assembly assays: method adapted from 25. 10 µM Vps1 (or mutant variant) was added to liposome buffer at the concentrations shown and centrifuged at 313,000g for 15 min to remove any aggregated protein. GTPγS was added to 0.5 mM, and oligomerized protein was pelleted by centrifugation at 250,000 g for 15 min, and supernatants and pellets analyzed by SDS-PAGE.

#### Liposome preparation

For preparation of liposomes 11 µl of a 25 mg/ml solution of Folch fraction 1 (Sigma) was dried under nitrogen, then resuspended in 200 µl of buffer B (20 mM HEPES pH 7.2, 100 mM KCl, 2 mM MgCl^2^, 1 mM DTT) at 60°C for 30 min with gentle agitation. Liposomes were extruded 11 times through polycarbonate filters with 1.0 µm pores.

#### Lipid binding assays

Vps1 (pre-spun at 313,000 g 15 min) was mixed with 20 µl liposomes to give a final concentration of 5 µM Vps1, in the presence of 2 mM GTP, 2 mM CaCl_2_, 2 mM MgCl_2_, and 2 mM DTT, and incubated at room temperature for 30 min. Liposomes and bound protein was pelleted by centrifugation at 250,000 g for 15 min, and samples were analyzed by SDS-PAGE. Alternatively, recombinant Vps1 was used at 1 µM to incubate with PIP strips and PIP arrays according to manufacturer’s instructions (Echelon). Binding was detected using anti-His antibodies (AbCam).

#### Electron microscopy

10 µl of 1 µM purified unspun Vps1 oligomers were visualized by negative staining. Samples were adsorbed on glow discharged carbon-coated copper grids and stained with 0.75 % uranyl formate. Electron micrographs were recorded on a Philips CM100 electron microscope using a Gatan MultiScan 794 CCD camera.

## References

[1] Konopka CA, Schleede JB, Skop AR, Bednarek SY (2006). Dynamin and cytokinesis.. Traffic.

[2] Praefcke GJ, McMahon HT (2004). The dynamin superfamily: Universal membrane tubulation and
fission molecules?. Nat Rev Mol Cell Biol.

[3] Schmid SL, Frolov VA (2011). Dynamin: Functional design of a membrane fission
catalyst.. Annu Rev Cell Devel Biol.

[4] Koenig JH, Ikeda K (1989). Disappearance and reformation of synaptic vesicle membrane upon
transmitter release observed under reversible blockage of membrane
retrieval.. J Neurosci.

[5] van der Bliek AM, Meyerowitz EM (1991). Dynamin-like protein encoded by the drosophila shibire gene
associated with vesicular traffic.. Nature.

[6] Achiriloaie M, Barylko B, Albanesi JP (1999). Essential role of the dynamin pleckstrin homology domain in
receptor-mediated endocytosis.. Mol Cell Biol.

[7] Vallis Y, Wigge P, Marks B, Evans PR, McMahon HT (1999). Importance of the pleckstrin homology domain of dynamin in
clathrin-mediated endocytosis.. Curr Biol.

[8] Morlot S, Roux A (2013). Mechanics of dynamin-mediated membrane fission.. Annu Rev Biophys.

[9] Urrutia R, Henley JR, Cook T, McNiven MA (1997). The dynamins: Redundant or distinct functions for an expanding
family of related GTPases?. Proc Natl Acad Sci U S A.

[10] Cocucci E, Gaudin R, Kirchhausen T (2014). Dynamin recruitment and membrane scission at the neck of a
clathrin-coated pit.. Mol Biol Cell.

[11] Durieux AC, Prudhon B, Guicheney P, Bitoun M (2010). Dynamin 2 and human diseases.. J Mol Med (Berl).

[12] Henley JR, Krueger EW, Oswald BJ, McNiven MA (1998). Dynamin-mediated internalization of caveolae.. J Cell Biol.

[13] Kreitzer G, Marmorstein A, Okamoto P, Vallee R, Rodriguez-Boulan E (2000). Kinesin and dynamin are required for post-golgi transport of a
plasma-membrane protein.. Nat Cell Biol.

[14] Liu YW, Surka MC, Schroeter T, Lukiyanchuk V, Schmid SL (2008). Isoform and splice-variant specific functions of dynamin-2
revealed by analysis of conditional knock-out cells.. Mol Biol Cell.

[15] Yang Z, Li H, Chai Z, Fullerton MJ, Cao Y, Toh BH, Funder JW, Liu JP (2001). Dynamin ii regulates hormone secretion in neuroendocrine
cells.. J Biol Chem.

[16] Bitoun M, Maugenre S, Jeannet PY, Lacene E, Ferrer X, Laforet P, Martin JJ, Laporte J, Lochmuller H, Beggs AH, Fardeau M, Eymard B, Romero NB, Guicheney P (2005). Mutations in dynamin 2 cause dominant centronuclear
myopathy.. Nat Genet.

[17] Dowling JJ, Gibbs EM, Feldman EL (2008). Membrane traffic and muscle: Lessons from human
disease.. Traffic.

[18] Zuchner S, Noureddine M, Kennerson M, Verhoeven K, Claeys K, De Jonghe P, Merory J, Oliveira SA, Speer MC, Stenger JE, Walizada G, Zhu D, Pericak-Vance MA, Nicholson G, Timmerman V, Vance JM (2005). Mutations in the pleckstrin homology domain of dynamin 2 cause
dominant intermediate charcot-marie-tooth disease.. Nat Genet.

[19] Boumil RM, Letts VA, Roberts MC, Lenz C, Mahaffey CL, Zhang ZW, Moser T, Frankel WN (2010). A missense mutation in a highly conserved alternate exon of
dynamin-1 causes epilepsy in fitful mice.. PLoS Genet.

[20] Patterson EE, Minor KM, Tchernatynskaia AV, Taylor SM, Shelton GD, Ekenstedt KJ, Mickelson JR (2008). A canine dnm1 mutation is highly associated with the syndrome of
exercise-induced collapse.. Nat Genet.

[21] Dhindsa RS, Bradrick SS, Yao X, Heinzen EL, Petrovski S, Krueger BJ, Johnson MR, Frankel WN, Petrou S, Boumil RM, Goldstein DB (2015). Epileptic encephalopathy-causing mutations in dnm1 impair
synaptic vesicle endocytosis.. Neurol Genet.

[22] Mears JA, Lackner LL, Fang S, Ingerman E, Nunnari J, Hinshaw JE (2011). Conformational changes in dnm1 support a contractile mechanism
for mitochondrial fission.. Nat Struct Mol Biol.

[23] Sesaki H, Southard SM, Yaffe MP, Jensen RE (2003). Mgm1p, a dynamin-related gtpase, is essential for fusion of the
mitochondrial outer membrane.. Mol Biol Cell.

[24] Nannapaneni S, Wang D, Jain S, Schroeder B, Highfill C, Reustle L, Pittsley D, Maysent A, Moulder S, McDowell R, Kim K (2010). The yeast dynamin-like protein vps1:Vps1 mutations perturb the
internalization and the motility of endocytic vesicles and endosomes via
disorganization of the actin cytoskeleton.. Eur J Cell Biol.

[25] Smaczynska-de Rooij II, Allwood EG, Aghamohammadzadeh S, Hettema EH, Goldberg MW, Ayscough KR (2010). A role for the dynamin-like protein vps1 during endocytosis in
yeast.. J Cell Sci.

[26] Vater CA, Raymond CK, Ekena K, Howaldstevenson I, Stevens TH (1992). The vps1 protein, a homolog of dynamin required for vacuolar
protein sorting in saccharomyces-cerevisiae, is a GTPase with 2 functionally
separable domains.. J Cell Biol.

[27] Wilsbach K, Payne GS (1993). Vps1p, a member of the dynamin GTPase family, is necessary for
Golgi membrane-protein retention in
Saccharomyces-cerevisiae.. EMBO J.

[28] Yu X, Cai M (2004). The yeast dynamin-related gtpase vps1p functions in the
organization of the actin cytoskeleton via interaction with
sla1p.. J Cell Sci.

[29] Durieux AC, Vassilopoulos S, Laine J, Fraysse B, Brinas L, Prudhon B, Castells J, Freyssenet D, Bonne G, Guicheney P, Bitoun M (2012). A centronuclear myopathy--dynamin 2 mutation impairs autophagy in
mice.. Traffic.

[30] Faelber K, Posor Y, Gao S, Held M, Roske Y, Schulze D, Haucke V, Noe F, Daumke O (2011). Crystal structure of nucleotide-free dynamin.. Nature.

[31] Ford MGJ, Jenni S, Nunnari J (2011). The crystal structure of dynamin.. Nature.

[32] Gu C, Yaddanapudi S, Weins A, Osborn T, Reiser J, Pollak M, Hartwig J, Sever S (2010). Direct dynamin-actin interactions regulate the actin
cytoskeleton.. EMBO J.

[33] Palmer SE, Smaczynska-de Rooij II, Marklew CJ, Allwood EG, Mishra R, Johnson S, Goldberg MW, Ayscough KR (2015). A dynamin-actin interaction is required for vesicle scission
during endocytosis in yeast.. Curr Biol..

[34] Reubold TF, Faelber K, Plattner N, Posor Y, Ketel K, Curth U, Schlegel J, Anand R, Manstein DJ, Noe F, Haucke V, Daumke O, Eschenburg S (2015). Crystal structure of the dynamin tetramer.. Nature.

[35] Raymond CK, Howald-Stevenson I, Vater CA, Stevens TH (1992). Morphological classification of the yeast vacuolar protein
sorting mutants - evidence for a prevacuolar compartment in class-e vps
mutants.. Mol Biol Cell.

[36] Robinson JS, Klionsky DJ, Banta LM, Emr SD (1988). Protein sorting in Saccharomyces-cerevisiae - isolation of
mutants defective in the delivery and processing of multiple vacuolar
hydrolases.. Mol Cell Biol.

[37] Hoepfner D, van den Berg M, Philippsen P, Tabak HF, Hettema EH (2001). A role for vps1p, actin, and the myo2p motor in peroxisome
abundance and inheritance in Saccharomyces cerevisiae.. J Cell Biol.

[38] Burston HE, Maldonado-Baez L, Davey M, Montpetit B, Schluter C, Wendland B, Conibear E (2009). Regulators of yeast endocytosis identified by systematic
quantitative analysis.. J Cell Biol.

[39] Valdez-Taubas J, Pelham HR (2003). Slow diffusion of proteins in the yeast plasma membrane allows
polarity to be maintained by endocytic cycling.. Curr Biol.

[40] Smaczynska-de Rooij II, Allwood EG, Mishra R, Booth WI, Aghamohammadzadeh S, Goldberg MW, Ayscough KR (2012). Yeast dynamin vps1 and amphiphysin rvs167 function together
during endocytosis.. Traffic.

[41] Chi RJ, Liu J, West M, Wang J, Odorizzi G, Burd CG (2014). Fission of snx-bar-coated endosomal retrograde transport carriers
is promoted by the dynamin-related protein vps1.. J Cell Biol.

[42] Smaczynska-de Rooij II, Marklew CJ, Allwood EG, Palmer SE, Booth WI, Mishra R, Goldberg MW, Ayscough KR (2015). Phosphorylation regulates the endocytic function of the yeast
dynamin-related protein vps1.. Mol Cell Biol..

[43] Barlow CA, Laishram RS, Anderson RA (2010). Nuclear phosphoinositides: a signaling enigma wrapped in a
compartmental conundrum.. Trends Cell Biol.

[44] Klein DE, Lee A, Frank DW, Marks MS, Lemmon MA (1998). The pleckstrin homology domains of dynamin isoforms require
oligomerization for high affinity phosphoinositide binding.. J Biol Chem.

[45] Kaiser C, Michaelis S, Mitchell A (1994). Methods in yeast genetics: A laboratory course
manual.. J Cell Biol.

[46] Motley AM, Hettema EH (2007). Yeast peroxisomes multiply by growth and
division.. J Cell Biol.

